# Crohn’s Disease Exclusion Diet for the Treatment of Crohn’s Disease: Real-World Experience from a Tertiary Center

**DOI:** 10.3390/jcm12165428

**Published:** 2023-08-21

**Authors:** Naomi Fliss-Isakov, Nathaniel Aviv Cohen, Ahuva Bromberg, Gal Elbert, Ronit Anbar, Yulia Ron, Ayal Hirsch, Tamar Thurm, Nitsan Maharshak

**Affiliations:** 1 Faculty of Medicine, Tel Aviv University, Tel Aviv 6997801, Israel; 2 Department of Gastroenterology and Liver Diseases, Tel Aviv Medical Center, Tel Aviv 6423906, Israel; 3 Nutrition and Dietetics Department, Tel Aviv Medical Center, Tel Aviv 6423906, Israel

**Keywords:** Crohn’s disease, dietary therapy, exclusion diet

## Abstract

The Crohn’s Disease (CD) exclusion diet (CDED) has been shown to induce remission in pediatric and adult patients with CD. In this retrospective cohort study, we describe our real-world experience with the CDED at the inflammatory bowel disease (IBD) unit of the Tel Aviv Medical Center between 2018–2021. CD patients with multiple clinical presentations and disease phenotypes who initiated the diet were included. Indications for treatment, medical and nutritional data were collected from dietician clinic visits and medical records. Clinical and biomarker responses were determined. The CDED was recommended to 220 CD patients. Seventy-two patients were included in the analysis for a clinically active disease (n = 48) or for remission maintenance (n = 24). Among patients with a clinically active disease, 62.5% of patients achieved clinical remission at week 6 and at week 12. A positive association between high adherence to the CDED and clinical remission at week 12 was observed (adjusted OR = 7.6, 95% CI 1.07–55.2, *p* = 0.043). Among patients treated for remission maintenance, remission at week 12 was maintained among 83.3% of patients. We conclude that the CDED may be a promising intervention for multiple CD presentations and indications. These findings should be further validated in larger, prospective, controlled studies.

## 1. Introduction

The incidence of Crohn’s disease (CD) has increased worldwide during the last few decades [1,2,3]. Recent advances have dramatically altered our appreciation of the importance of diet in CD [4,5] and support the association between diet, and specifically ultra-processed foods, with CD development and progression [6,7]. Multiple pathways have been described to support epidemiologic data, including diet’s effect on the composition and function of the microbiome, intestinal barrier, and immune response [8,9].

The CD exclusion diet (CDED) is a whole-food diet designed to reduce exposure to dietary components hypothesized to be detrimental to the gut microbiome, intestinal barrier, and intestinal immunity while maintaining a balanced diet and the patient’s nutritional status [10]. The mandatory foods provide specific fibers and starches as substrates for short-chain fatty acids–producing bacteria belonging to the Firmicutes phylum [11,12]. Most importantly, the CDED mandates avoidance of foods rich in animal/dairy fat, wheat, red or processed meat, protein sources rich in taurine, and dietary additives such as emulsifiers, artificial sweeteners, carrageenan, and sulfites, all of which have been previously shown to be associated with gut inflammation in various models [10,13,14,15,16]. Indeed, clinical responses following CDED have been associated with changes in microbial composition and function [17,18].

The CDED has been previously shown to induce remission and decrease objective markers of inflammation in pediatric populations [17,19,20,21]. Recently, we have shown that the CDED, either with or without concomitant partial enteral nutrition (PEN), can also induce remission in adult patients [22]. These studies included a very specific population of patients suffering from an uncomplicated, inflammatory, short (<5 years), mild-to-moderate disease involving only the terminal ileum. However, CDED can potentially serve as a therapeutic option for a wider range of CD presentations and can be combined with advanced therapies as a therapeutic strategy [23]. Though the CDED is not widely recommended, the positive reports of it’s outcomes have led us to include it as a therapeutic option for patients with CD, in our tertiary referral center for patients with inflammatory bowel disease (IBD). In this observational study, we describe our real-world experience with the CDED in CD.

## 2. Materials and Methods

### 2.1. Study Design

A retrospective cohort study was performed at the IBD unit of the Tel Aviv Medical Center (TLVMC) between January 2018 and November 2021. The study was approved by the local ethics committee of the TLVMC. Patient consent was waived due to the retrospective observational nature of the study.

### 2.2. Study Population

We collected data on all CD patients who were referred to treatment with the CDED by their treating physician. The following criteria excluded patients from the study: lack of documentation of physician’s indication for dietary therapy, previous experience with the CDED, patient’s unwillingness to adhere to the diet, or if carrying a stoma.

### 2.3. Data Collection

All data were collected from medical records from our multidisciplinary clinic, in which patients are treated by their physician, nurse, and dietician. All visits (frontal, telephone, or correspondence) were documented in one clinical data management system. Data collection was performed in a systematic manner according to a single data collection protocol by one observer, a registered dietician who is highly familiar with the CDED treatment protocol [24]. The principles of the CDED have been described elsewhere [19,20,21]. Briefly, the CDED is a whole-food diet coupled with PEN, designed to reduce exposure to dietary components, hypothesized to negatively affect the microbiome (dysbiosis), intestinal barrier, and intestinal immunity. The diet included five mandatory foods consumed daily to provide specific fibers and starches as substrates for short-chain fatty acids–producing taxa from Firmicutes, as well as sources of lean protein that were low in animal fat to decrease Proteobacteria and improve intestinal permeability while maintaining a balanced diet. The diet included avoidance or reduction of exposure to foods containing animal/dairy fat, high fat from other sources, wheat, red or processed meat, and protein sources rich in taurine, emulsifiers, artificial sweeteners, carrageenans, and sulfites. The second phase stepdown diet involves higher exposure to fruits, vegetables, and legumes, along with some foods that are reintroduced with restrictions to increase food flexibility and relieve monotony [19,20,21]. Patients’ medical backgrounds, medical therapy, clinical and biomarker disease activity, dietary instructions, and patients’ adherence were documented before and throughout the three phases of the diet.

Indications for the CDED were categorized into one of the following: ‘patient’s will’—patients who had other options but insisted on CDED or patients who were in clinical remission but had mild symptoms not justifying a formal therapy, ‘adjunctive therapy’—an add-on to another therapy in case of partial response, ‘bridging intervention’—during biologic therapy induction until achieving response, ‘rescue therapy’—in patients losing response to another therapy, ‘salvage therapy’—in case of exhaustion of advanced therapeutic options, or contraindication to advanced therapeutic options, or ‘pre-surgical intervention’—in cases of preparation for surgery.

Active disease was defined by at least one of the following: clinical disease activity [Harvey Bradshaw Index (HBI) ≥ 5], biomarker disease activity [Fecal Calprotectin (Fcal) ≥250 mg/kg] or endoscopic disease activity [Simple Endoscopic Score-CD (SES-CD) ≥ 7 or Rutgeerts score ≥ i2] [25,26]. Patients’ disease activity was documented at baseline, week 6 (end of phase 1), week 12 (end of phase 2, end of induction), and week 24 (phase 3). In patients where HBI was not documented at week 6, but a physician global assessment was documented as “non-response to therapy” (n = 6), or in patients who were lost to follow-up by week 12, HBI at week 12 was imputed (last observation carried forward).

Clinical and biomarker outcomes were calculated among all patients, as the difference between values at baseline and week 12 (baseline-week 12). Clinical response was defined as a decrease of ≥3 points in HBI, and clinical remission was defined as HBI < 5 points among those who suffered from a clinically active disease at diet initiation [25].

Nutritional status and needs were documented by treating dieticians during clinic visits and were based on body mass index (BMI), weight loss history, and dietary intake at baseline. The malnutrition universal screening tool (MUST) score was documented and used to assess malnutrition development risk at every clinic visit. Patient’s dietary pattern before the diet, nutritional adaptations to the CDED (for comorbidities such as diabetes, osteoporosis, etc., a stricturing/post-surgical disease, nutrient deficiencies, pregnancy, remission, etc.), or personal preferences (such as vegetarianism/veganism), were all documented in a standardized manner. The use of PEN was instructed according to the dietician’s considerations in some but not in all patients.

The last phase of the diet, which was completed by each patient (week 6/12/24/ > 24 weeks), and the duration of follow-up (weeks) were documented. During dietician’s clinic visits, a 24-h dietary recall is routinely obtained and documented. Adherence to the diet on a 5-level Likert scale [27] was reported during patients’ follow-up visits to the clinic by the dieticians or physicians (Not compliant/Non-compliant but willing/Partially compliant/Fairly compliant/Very compliant). High adherence to the diet was defined as fairly/very adherent. 

### 2.4. Statistical Analysis

All statistical analyses were performed using SPSS version 25.0 for Windows (SPSS Inc., Chicago, IL, USA). Continuous variables are presented as means ± SD and nominal variables as proportions. Pearson’s Chi-Square test was used to test the association between nominal variables. Comparison of continued variables between study groups was performed by the independent samples *t*-test for variables that distributed normally (BMI and SES-CD) and by the Mann–Whitney test for variables that did not distribute normally. The difference in continuous variables throughout follow-up visits was assessed using the dependent sample *t*-test for BMI or the Wilcoxon matched pair signed-rank test. Logistic regression was used to identify adjusted associated factors of diet-induced remission.

## 3. Results

### 3.1. Study Population

**Figure 1:**
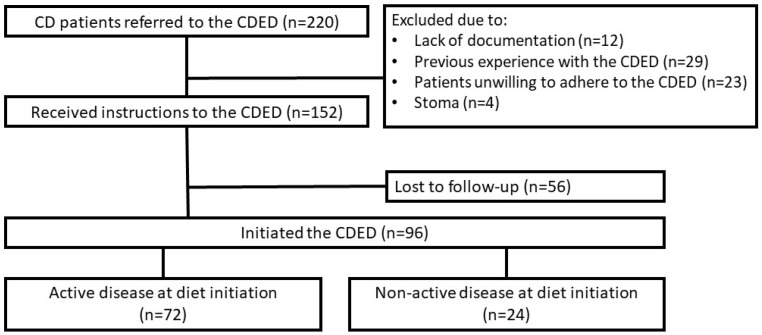
Crohn’s disease study population flowchart. Legend: Active disease was defined as either clinical (HBI ≥ 5), biomarker (Fcal ≥ 250 mg/kg), or endoscopic disease activity (SES-CD ≥ 7 or Rutgeerts score ≥ i2). Abbreviations: CD—Crohn’s Disease, CDED—Crohn’s disease exclusion diet.

The CDED was recommended to 220 CD patients, who were referred to a dietitian’s consultation, mostly due to their own will to be treated by a non-pharmacologic modality (45.0%). Additional common indications included—adjuvant therapy (26.0%) and a bridging intervention (20.0%) (Supplementary Figure S1). After the exclusion of ineligible patients, a total of 152 CD patients received instructions for the CDED, of whom 56 (36.8%) patients did not return to follow-up after the initial consultation (Figure 1).

Patients who did not initiate the CDED were significantly older and had higher BMI but did not differ otherwise from patients who initiated the CDED (Supplementary Table S1).

Eventually, 96 patients followed the diet: 72 patients due to an active disease [clinical (HBI ≥ 5, n = 48), biomarker (Fcal ≥ 250 mg/kg, n = 19) and/or endoscopic (SES-CD ≥ 7 or Rutgeerts score ≥ i2, n = 5)] and 24 patients with an inactive disease, to maintain remission (Figure 1, Table 1).

### 3.2. Clinical Improvement following the CDED

Among patients with an active disease (clinical, biomarker, or endoscopic) 45.8% (33/72) achieved clinical response. Patient and disease characteristics, associated with week-12 clinical response is depicted in Supplementary Table S2.

Among patients with clinically active disease, 62.5% (30/48) achieved clinical remission by week-6; 62.5% were in remission by week-12, and 16.7% (8/48) patients were in remission by week-24. Between weeks 6 and 12, only one patient lost response, and another patient went into remission.

**Figure 2:**
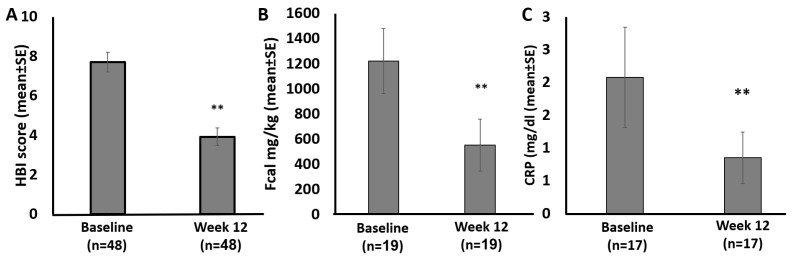
Clinical and biomarker improvement following the CDED. HBI improved significantly in patients who initiated the diet with a clinically active disease (**A**). Fcal improved significantly in patients who initiated the diet with Fcal ≥ 250 mg/kg (**B**). CRP improved significantly in patients who initiated the diet with CRP ≥ 0.5 mg/dl (**C**). ** *p* < 0.001. Abbreviations: CRP—C-reactive protein, Fcal—fecal calprotectin, HBI—Harvey Bradshaw Index.

Among patients with clinically active disease, HBI decreased by an average of 3.7 ± 3.5 (n = 48), and among patients with biomarker active disease, Fcal decreased by 668 ± 1284 mg/kg (n = 19) by week 12 of the CDED (Figure 2).

**Figure 3:**
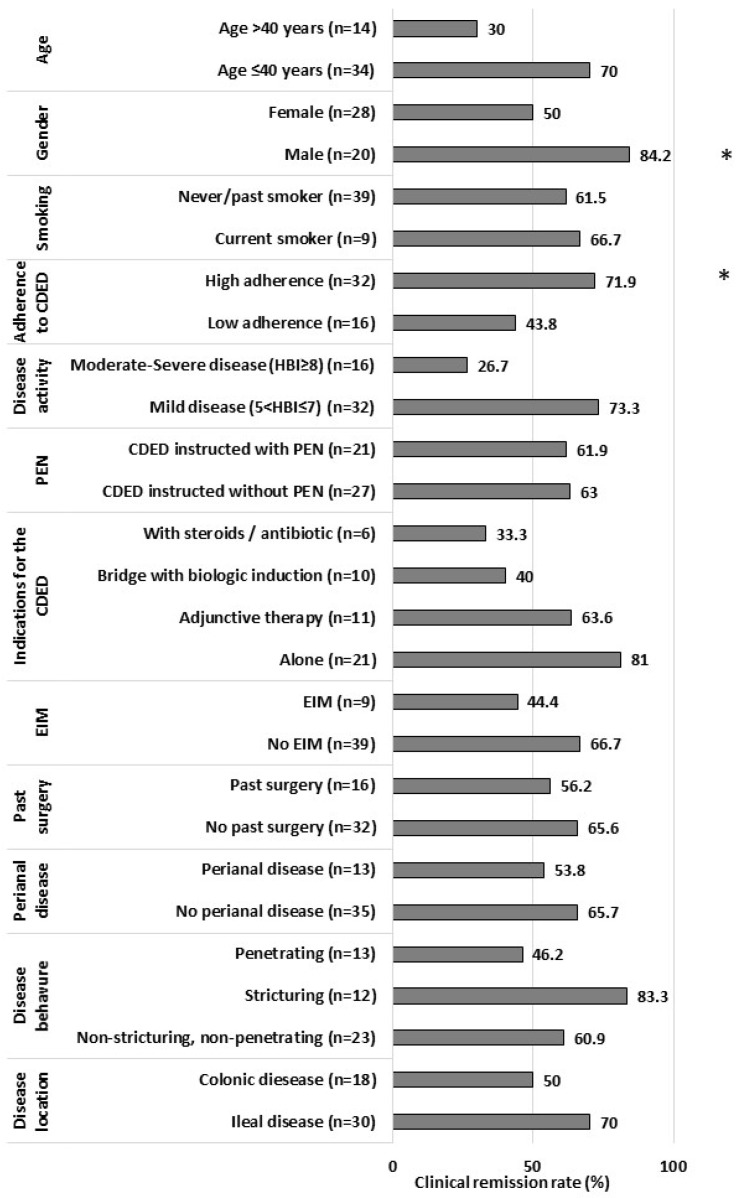
Clinical remission rate at week 12 across patient and disease characteristic subgroups (n = 48). * *p* < 0.05. Abbreviations: EIM—extra-intestinal manifestations, CDED—Crohn’s disease exclusion diet, PEN—Partial enteral nutrition.

Male gender, BMI, and adherence to the CDED were positively associated with week-12 clinical remission (Table 2, Figure 3), while demographics, disease phenotype, biologic treatment, and adaptations to the diet were not. In a multivariate analysis, there was a positive association between high adherence to the CDED and clinical remission in week 12, when adjusting to age, gender, disease duration, BMI, and baseline HBI score (OR = 7.6, 95% CI 1.07–55.20, *p* = 0.043).

Patients who were treated with the CDED alone for a clinically active disease, compared to those receiving CDED with other interventions, had significantly higher remission rates at the end of week 6 and at the end of induction (81.0% vs. 48.1%, Pv = 0.020) and were characterized by higher proportions of an ileal disease (Pv = 0.018), a non-stricturing non-penetrating disease (Pv = 0.001), and lower proportions of a perianal disease (Pv = 0.002), and surgical history (Pv = 0.014) (Supplementary Table S3).

### 3.3. CDED Is Effective to Maintain Remission

Among the patients who were treated with CDED to maintain remission, remission at week 12 was maintained among 83.3% (20/24) of patients. Moreover, 33.3% (8/24) experienced a reduction in HBI score of 0.6 ± 1.1 points (range 1–3 points). High adherence to the diet was documented among 66.7% of patients. Only 20.8% (5/24) of the patients eventually required an additional treatment within 6.8 ± 6.5 months of CDED initiation.

### 3.4. Adaptations, Adherence, and Safety to the CDED

Among the 96 patients who were followed, the mean length of follow-up was 14.8 ± 9.4 weeks (range 3–36 weeks). Among patients with clinically active disease, 52.1%, 37.5%, and 6.3% of patients completed 6, 12, and 24 weeks of the diet, respectively. Only 4.2% of patients continued dietary follow-up for a longer period of time (Supplementary Table S4). Most of the patients were highly adherent (n = 54, 56.9%), 20% were partially adherent, 23.1% were not adherent, and only three patients reported intolerance to the diet due to palatability. Compared with patients who were highly adherent to the diet, partially/non-adherent patients were younger (32.7 ± 13.7 vs. 40.5 ± 16.9, *p* = 0.043) and had lower BMI (21.5 ± 3.8 vs 23.5 ± 3.4, *p* = 0.033). There was no difference between these groups in gender (Pv = 0.277), disease duration (Pv = 0.328), clinical disease activity (Pv = 0.999), biomarker disease activity (Pv = 0.891), surgical history (Pv = 0.350), or indication for PEN (Pv = 0.350).

**Figure 4:**
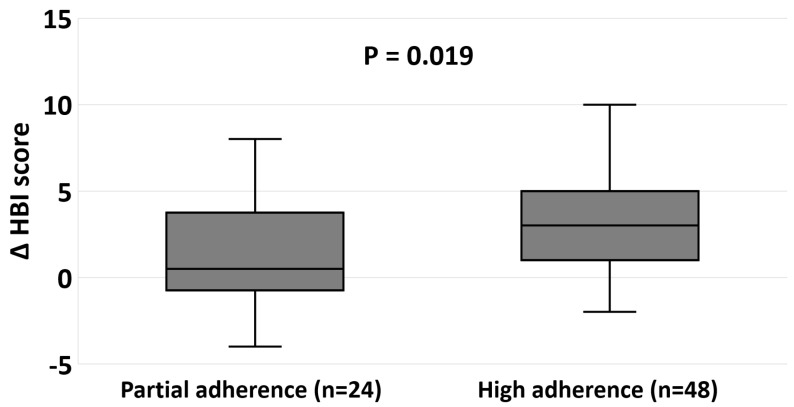
Clinical improvement at week 12 by adherence to the CDED among patients with active disease at diet initiation (n = 72). High adherence to the CDED was defined as very/fairly adherent. ΔHBI score is the difference between HBI scores at baseline and week 12 (baseline-week 12). Abbreviations: HBI—Harvey Bradshaw Index.

In patients who initiated the diet with an active disease (clinical/biomarker/endoscopic) (n = 72), high adherence to the CDED, reported among 66.7% of patients, was associated with a higher decrease in HBI at week-12 (Figure 4).

To accommodate the diet to patients’ clinical characteristics and preferences, dieticians made several adaptations to the CDED, such as a low-fiber adaptation for intestinal strictures or post-operative patients (23.0%), commencing CDED directly at phase 2/3 for patients in remission/mild disease (16.5%), micronutrient supplementation (10.5%), plant-based adaptations for vegetarian/vegan patients (3.9%), low fermentable oligosaccharides, disaccharides, monosaccharides and polyols (FODMAPs) adaptations (2.6%). In no case were patients instructed to consume CDED-prohibited foods. Treatment with PEN was associated with poor nutritional status (*p* < 0.001) and not with disease activity (*p* = 0.144). High adherence to PEN was documented in 43.3% of the patients.

The mean BMI at diet initiation and at the end of follow-up was 23.0 ± 4.4 kg/m^2^, and 22.5 ± 3.7 kg/m^2^, respectively (mean drop of 0.3 ± 1.4 kg/m^2^). Twelve patients (16.6%) were underweight (BMI ≤ 18.5 kg/m^2^) at baseline and remained underweight at the end of follow-up (n = 7) or achieved a normal weight (BMI ≥ 18.5 kg/m^2^) (n = 5). Interestingly, patients treated with CDED + PEN gained an average of 0.3 ± 1.4 kg/m^2^ compared to patients who received only CDED, who lost on average 0.6 ± 1.3 kg/m^2^ (*p* = 0.005). Seven patients (9%) lost a significant amount of weight during follow-up (mean loss of 2.3 ± 1.2 BMI units).

## 4. Discussion

In this real-life retrospective study, the CDED was found to be effective in improving clinical and biomarker activity in multiple CD presentations and indications that were previously excluded from clinical trials. CDED was prescribed as a bridge therapy during preparation for advanced therapy, as an adjunctive therapy for patients who do not completely respond to therapy, or, most commonly, in patients who suffer from very mild symptoms that do not necessarily justify advanced or immune-modulator therapy. Interestingly, the most common indication for the CDED in our cohort was the patient’s will, underscoring the importance of dietary interventions in empowering patients and improving adherence to a treatment plan.

Although the pathogenesis of CD remains to be fully elucidated, environmental factors such as diet are believed to play a pivotal role in the onset and management of CD [28]. To date, the dietary therapy most studied, and the only dietary therapy, which is widely recommended for remission induction in CD, is EEN. A recent meta-analysis reported remission rates ranging from 20% to 84.2% following EEN [29]. The CDED is a whole-food dietary strategy that has been proposed as an alternative to EEN [30]. Levin et al. performed a 12-week randomized clinical trial (RCT) among children with mild-moderate CD, showing a remission rate of 75.0% following CDED + PEN and 59.0% following EEN by week 12 [17]. The CDED + PEN was later compared to the CDED alone in a multi-center RCT, among biologically naïve adult CD patients, with an early, mild-moderate disease. The remission rate was 68.0% in the CDED + PEN group and 57.0% in the CDED group at week 6, and was highly associated with remission at week 12 [22]. Indeed, these studies suggest that the CDED may be effective in inducing remission in CD patients, though their results and conclusions are limited to certain disease phenotypes/characteristics included in these studies. Other whole-food dietary modalities studied in CD reported similar results. The effects of an individualized food-based diet for mild-moderate CD (CD-TREAT) among 25 patients achieved a 60% remission rate and a significant reduction in Fcal after 6 weeks in a pediatric population [31]. A large RCT comparing the specific carbohydrate diet (SCD) and the Mediterranean diet for the treatment of active mild-moderate CD in adults resulted in a week 6 remission rate of 46.5% and 43.5%, respectively [32]. Two other diets currently investigated in the clinical trial setting are the ‘Tasty & Healthy’ dietary approach, which excludes pro-inflammatory ingredients but allows full flexibility of the allowed foods (Clinicaltrials.gov NCT04239248), and the Anti-Inflammatory Diet that restricts certain carbohydrates, includes prebiotics and probiotics, and modifies dietary fatty acids [33].

Two recently published articles describe real-world experiences with the CDED. A study by Szczubełek et al. described the results of the CDED + PEN in 32 adult patients with CD. A high remission rate of 76.7% was reported after 6 weeks and 82.1% after 12 weeks, which might be explained by the exclusion of a wide array of patients with severe disease [34]. In a retrospective study by Niseteo et al., data were extracted from the medical records of 61 children with CD who were treated with CDED + PEN or EEN. Remission was achieved by 68.9% among patients at 6 weeks, with no difference between groups [35].

In the current observational study, clinical remission after 6 and 12 weeks of the diet was documented among 62.5% of the participants who suffered from an active disease at diet initiation. Remission rates were higher among patients who were treated with the CDED alone, potentially due to a more favorable disease phenotype and location- a non-structuring non-penetrating disease involving the ileum, with less perianal disease involvement and a lower percentage of surgical history. Nonetheless, although we prescribed CDED to a wide range of clinical phenotypes and disease presentations, all previously excluded from the prospective CDED clinical trial [22], the overall 12-week remission rates are comparable. Nevertheless, the week 24 remission rate of 16.7% in our current study was lower, probably underscoring the importance of a long-term follow-up and support by a dietitian who is more available in clinical trials. Remission rates in this study population, as in the CDED-AD trial [22], were lower than that reported among pediatric CD patients [17,19,20]. This may be attributed to adherence to the diet, which has been reported to be higher in children relative to adults [36].

Not surprisingly, CDED was very effective in maintaining remission. Patients in clinical remission present an unmet therapeutic need, as most of these patients suffered from mild symptoms not justifying the addition of immune-modulators or advanced therapies. Interestingly, they demonstrated high adherence for at least 12 weeks, and a third demonstrated additional improvement in clinical symptoms.

The CDED, particularly during its early stages, may lead to weight loss, risk of malnutrition, and micronutrient deficiency, if not properly controlled [37]. Indeed, three of the patients (4%) who lost weight throughout the diet in our cohort were defined as underweight at follow-up. Other than calcium and vitamin D, which are not provided by the whole foods of the diet and are supplemented per protocol, the diet reaches the nutrient needs of a standalone diet. Nevertheless, the risk for malnutrition should be taken into account, especially in cases with baseline restrictive eating due to severe symptoms, other co-morbidities associated with a restricting diet, vegetarianism, and in cases of increased nutritional needs such as pregnancy and lactation [38].

Importantly, we did not observe any differences in remission rates following the CDED among subgroups of CD phenotype, disease location, extra-intestinal activity, therapeutic strategy, disease duration, or age. Furthermore, no significant differences were detected between patients treated with or without PEN, an option that might be more attractive to some patients. This finding is in accordance with previous results demonstrating that CDED with or without PEN was equally effective for induction and maintenance of remission in adults with CD [22]. We found higher remission rates among males and patients with high adherence to therapy and lower remission rates in patients with higher baseline clinical disease activity and treated with steroids. These results are in agreement with previous reports of low adherence to the CDED being associated with reduced odds of remission at week 6 [21]. Patients with higher clinical activity might have a lower baseline intake of dietary fiber and plant-based food, which have been found to be beneficial in changing patients’ microbiome activity [12].

Even though 36.8% of patients who received instructions for the CDED were lost to follow-up, most patients were able to maintain it for at least 12 weeks with high adherence rates. These estimates might be attributed to the fact that the CDED comprises all food groups and is diverse relative to other restrictive elimination diets [39,40,41,42], which may be more difficult to adhere to [43]. They are independently associated with poorer food-related quality of life and reduced appetite, and possibly limit adherence [44].

The limitations of this study include the potential information bias of a retrospective study. Further, food diaries were not available to assess adherence. This bias was minimized by a meticulous and standardized data collection and validation protocol. Data were gathered by one observer in the same manner to prevent differential information bias. Adherence to the CDED was evaluated based on highly informative 24-h recall assessments documented throughout clinic visits. This enabled the assessment of adherence to the diet in a standardized and objective manner. Further, given that the CDED is quite restrictive in its first two phases, and patients usually reach a repetitive eating pattern, we assume this method has only a minor potential for information bias. A non-differential misclassification bias of the diet effects may have existed, given that remission was determined based on clinical parameters without sufficient biomarkers and endoscopic results. Additionally, loss to follow-up is a major limitation of this observational study, as anticipated in a real-world setting. Consequently, statistical power is limited and limits firm conclusions regarding each of the indications, clinical phenotypes, or treatment strategies of the patients due to the small number of patients in each group. Importantly, since the CDED was not compared to another dietary strategy, we cannot conclude it is more effective than any other diet. Last, our study population is patients treated in a tertiary hospital, limiting the generalizability of results due to potential referral filter bias.

Strengths of our study include a relatively large study population which was followed until the end of the CDED induction phase, with diverse demographic and medical characteristics, thereby minimizing potential selection bias and residual confounding. Most importantly, this study showed that CDED may be applicable and helpful in a wide range of CD phenotypes and conditions.

## 5. Conclusions

This study elaborates on the observed effect of the CDED, a whole-food diet, for the treatment of CD. Here we demonstrated high remission rates among patients of a wide array of disease and treatment characteristics. Our results imply that patients who may most benefit from the CDED are those who would adhere to the diet. These findings should be further validated in a prospective larger, controlled study.

## Figures and Tables

**Table 1:** Patient and disease characteristics at diet initiation, and a comparison between patients who initiated the diet with active disease to patients in remission.

	Active Disease (n = 72)	Inactive Disease (n = 24)	*p*
Demographic characteristics			
Age (years, mean ± std)	35.3 ± 15.2	37.8 ± 16.9	0.504
Gender—female n, (%)	41, (56.9)	14, (58.3)	0.960
Ever smoker n, (%)	21, (29.2)	0, (0)	0.132
Family history of IBD n, (%)	12, (16.7)	6, (25)	0.065
Disease duration (years, mean ± std)	7.0 ± 8.0	10.3 ± 13.9	0.258
BMI (kg/m^2^, mean ± std)	22.2 ± 3.7	23.6 ± 4.1	0.143
CD Montreal phenotype n, (%)			
A1—Below 16 years	15, (20.8)	6, (25.0)	0.754
A2—Between 17 and 40 years	40, (55.6)	10, (41.7)	
A3—Above 40 years	17, (23.6)	8, (33.3)	
L1—Ileal	46, (63.9)	19, (79.2)	0.304
L2—Colonic	3, (4.2)	0, (0)	
L3—Ileo-colonic	23, (31.9)	5, (20.8)	
L4—Proximal disease	10, (13.9)	2, (8.3)	0.476
B1—Non-stricturing, non-penetrating	32, (44.4)	13, (54.2)	0.631
B2—Stricturing	18, (25.0)	4, (16.7)	
B3—Penetrating	22, (30.6)	7, (29.2)	
Perianal disease	16, (22.2)	7, (29.2)	0.490
Past surgery n, (%)	23, (31.9)	7, (29.2)	0.405
Extra-intestinal manifestations n, (%)	11, (15.3)	5, (20.8)	0.527
Biologic therapy experience n, (%)			
Naïve	39, (54.2)	11, (45.8)	0.306
Past therapy	4, (5.6)	0, (0)	
Current therapy	29, (40.3)	13, (54.2)	
Disease activity at diet initiation			
HBI (mean ± std)	5.7 ± 4.0	1.6 ± 1.5	<0.001
CRP (mg/dL, mean ± std)	1.9 ± 2.2 (n = 57)	1.8 ± 2.8 (n = 18)	0.910
Fcal (mg/kg, mean ± std)	765 ± 1140 (n = 53)	115 ± 74 (n = 12)	0.054
SES-CD score (mean ± std)	7.0 ± 4.6 (n = 25)	5.0 ± 1.4 (n = 2)	0.555
Rutgeerts score (mean ± std)	2.0 ± 1.5 (n = 11)	1.0 (n = 1)	0.506
CDED indications and adaptations n, (%)			
CDED alone	29, (40.3)	10, (41.7)	0.187
Adjunctive therapy	17, (23.6)	6, (25.0)	
Bridge therapy	18, (25.0)	2, (8.3)	
With steroids/antibiotics	8, (11.1)	6, (25.0)	
PEN prescribed with CDED n, (%)	22, (30.5)	5, (20.8)	0.204

Abbreviations: CD—Crohn’s disease, BMI—body mass index, CRP—C-reactive protein, Fcal—fecal calprotectin, SES-CD—simple endoscopic score for CD, HBI—Harvey Bradshaw Index.

**Table 2:** Patients’ and disease characteristics at baseline in patients with clinically active disease according to week 12 clinical status.

	Clinical Remission ^a^ (n = 30)	No Clinical Remission (n = 18)	*p*
Demographic characteristics			
Age (years, mean ± std)	37.7 ± 17.3	33.2 ± 15.4	0.376
Gender—male n, (%)	16, (53.3)	4, (22.2)	0.017
Ever smoker n, (%)	6, (20.0)	3, (16.7)	0.775
Disease duration (years, mean ± std)	7.4 ± 9.1	6.8 ± 8.4	0.823
BMI (kg/m^2^, mean ± std)	23.1 ± 4.3	20.7 ± 2.7	0.029
Disease characteristics at baseline n, (%)			
A1—Below 16 years	5, (16.7)	3, (16.4)	0.938
A2—Between 17 and 40 years	17, (56.7)	11, (61.1)	
A3—Above 40 years	8, (26.7)	4, (22.2)	
L1—Ileal	21, (70.0)	9, (50.0)	0.218
L2—Colonic	1, (3.3)	0, (0.0)	
L3—Ileo-colonic	8, (26.7)	9, (50.0)	
L4—Proximal disease	5, (16.7)	3, (16.7)	1.000
B1—Non-stricturing, non-penetrating	14, (46.7)	9, (50.0)	0.550
B2—Stricturing	10, (33.0)	2, (11.1)	
B3—Penetrating	6, (20.0)	7, (38.9)	
Perianal disease	7, (23.3)	6, (33.3)	0.450
Past surgery	9, (30.0)	7, (58.9)	0.527
Extra-intestinal manifestations	4, (13.3)	5, (27.8)	0.215
Medical treatment n, (%)			
Naïve	19, (63.3)	8, (44.4)	0.266
Past therapy	1, (3.3)	0, (0.0)	
Current therapy	10, (33.3)	10, (55.6)	
Disease activity			
HBI (mean ± std)	6.9 ± 2.3	9.0 ± 4.1	0.071
CRP (mg/dL, mean ± std)	2.0 ± 1.7	2.0 ± 2.7	0.553
Fcal (mg/kg, mean ± std)	315 ± 376	862 ± 1253	0.168
Biomarker active disease n, (%) ^b^	8, (26.7)	9, (50.0)	0.102
SES-CD score (mean ± std)	8.1 ± 4.9	7.0 ± 5.0	0.643
Rutgeerts score (mean ± std)	2.0 ± 2.0	1.0 ± 1.7	0.700
Endoscopic active disease n, (%) ^c^	7, (23.3)	6, (33.3)	0.450
Indications and adaptations for the CDED n, (%)			
CDED alone	17, (56.7)	4, (22.2)	0.060
Adjunctive therapy	7, (23.3)	4, (22.2)	
Bridge therapy	4, (13.3)	6, (33.3)	
With steroids/antibiotic	2, (6.7)	4, (22.2)	
PEN, prescribed with CDED n, (%)	13, (43.3)	8, (44.3)	0.940
High adherence to the CDED ^d^	23, (76.7)	9, (50.0)	0.058

^a^ Clinical remission was defined as HBI < 5 points ^b^ Biomarker disease activity was defined as Fcal ≥ 250 mg/kg d ^c^ Endoscopic disease activity as defined as either SES-CD ≥ 7 or Rutgeerts score ≥ i2 ^d^ High adherence to the diet was defined as fairly/very adherence.

## Data Availability

Data available on request due to restrictions, e.g., privacy or ethical.

## Supplementary Materials

The following supporting information can be downloaded at: https://www.mdpi.com/article/10.3390/jcm12165428/s1, Figure S1: Indications for referral to the CDED; Table S1: Demographic and disease characteristics of patients who received instructions for the CDED; Table S2: Patient and disease characteristics associated with week 12 clinical response. Table S3: Demographic and disease characteristics of patients with a clinically active disease allocated to the CDED alone compared to those treated with the CDED on top of other treatment options (n = 48). Table S4: Demographic and disease characteristics of patients with a clinically active disease by follow-up duration with the CDED (n = 48).
