# Supplementary material for: Crohn’s Disease Exclusion Diet for the Treatment of Crohn’s Disease: Real-World Experience from a Tertiary Center
Source: J Clin Med. 2023 Aug 21;12(16):5428. doi: 10.3390/jcm12165428 (PMC10455757; doi:10.3390/jcm12165428)
Supplement: Supplementary file 1 [file jcm-12-05428-s001.zip › jcm-2516330-supplementary.pdf]

Supplementary Figure S1. Indications for referral to the CDED (N=220)

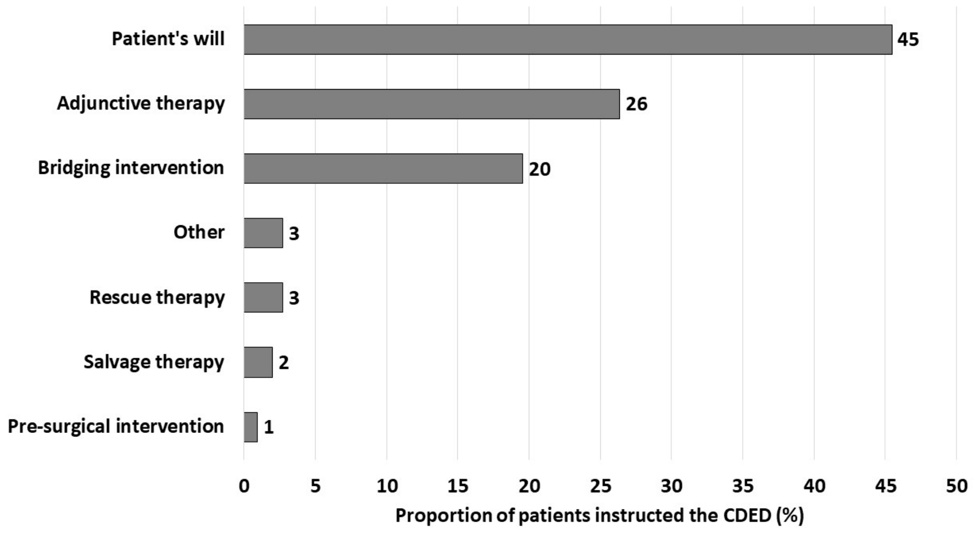

Supplementary Table S1. Demographic and disease characteristics of patients who received instructions for the CDED and initiated it, compared to those who did not initiate the diet.

	All patients N=152	Initiated CDED (n=96)	Did not initiate CDED (n=56)	P
Demographic characteristics				
Age (years, mean±std)	38.0±16.3	36.0±15.6	41.6±17.0	0.042
Gender – female n, (%)	64, (42.1)	40, (42.1)	24, (42.9)	0.928
Ever smoker n, (%)	52, (34.2)	34, (35.4)	18, (31.1)	0.141
Disease duration (years, mean±std)	8.0±9.7	7.8±9.6	8.3±9.8	0.757
BMI (kg/m^2, mean±std)	23.3±5.1	22.5±3.9	24.6±6.5	0.018
CD Montreal phenotype n, (%)				
A1 - Below 16 years	31, (20.4)	21, (21.9)	10, (17.9)	0.179
A2 - Between 17 and 40 years	80, (52.6)	54, (56.3)	26, (46.4)	
A3 - Above 40 years	41, (27.0)	21, (21.9)	20, (35.7)	
L1 - Ileal	97, (63.8)	65, (67.7)	32, (57.1)	0.419
L2 - Colonic	5, (3.3)	3, (2.0)	2, (1.3)	
L3 - Ileo-colonic	50, (32.9)	28, (29.2)	22, (39.3)	
L4 - Proximal disease	22, (14.5)	12, (12.5)	10, (18.2)	0.341
B1 - Non-stricturing, non-penetrating	78, (51.3)	45, (46.9)	33, (58.9)	0.212
B2 - Stricturing	35, (23.0)	22, (22.9)	13, (23.2)	
B3 - Penetrating	39, (25.7)	29, (30.2)	10, (17.9)	
Perianal disease	35, (23.0)	23, (24.0)	12, (21.4)	0.721
Past surgery n, (%)	43, (28.3)	30, (31.2)	13, (23.3)	0.509
Extra-intestinal manifestations n, (%)	29, (19.1)	16, (16.7)	13, (23.2)	0.322
Biologic therapy experience n, (%)				
Naïve	79, (52.0)	50, (52.1)	29, (52.7)	0.134
Past therapy	10, (6.6)	4, (4.2)	6, (10.9)	
Current therapy	62, (40.7)	42, (35.4)	20, (36.4)	
Disease activity at diet initiation				
HBI (mean±std)	5.0±4.0	4.7±3.9	5.5±4.0	0.224
CRP (mg/dl, mean±std)	2.4±4.5 (n=118)	1.9±2.3 (n=75)	3.2±6.8 (n=43)	0.135
Fcal (mg/kg, mean±std)	594±1034 (n=102)	645±1059 (n=65)	505±998 (n=37)	0.514
SES-CD score (mean±std)	6.7±4.5 (n=46)	6.8±4.4 (n=27)	6.6±4.7 (n=19)	0.904
Rutgeerts score (mean±std)	2.0±1.4 (n=17)	2.0±1.4 (n=12)	2.2±1.4 (n=5)	0.803
Active disease [a] n, (%)	111, (73.0)	72, (75.0)	39, (69.6)	0.576

[a] Active disease was defined by at least one of the following: clinical disease activity (HBI≥ 5, biomarker disease activity (Fcal≥250 mg/kg) or endoscopic disease activity (SES-CD ≥ 7 or Rutgeerts score ≥ i2).

Abbreviations: CD - Crohn's disease, BMI – body mass index, CRP – C-reactive protein, Fcal – fecal calprotectin, SES-CD – simple endoscopic score for CD, HBI – Harvey Bradshaw Index

Supplementary Table S2. Patient and disease characteristics associated with week-12 clinical response (Patients with active disease, n=72)

	Clinical response [a] (n=33)	No clinical response (n=39)	P
Demographic characteristics			
Age (years, mean±std)	35.2±16.3	35.4±14.5	0.750
Gender – male n, (%)	13, (40.6)	17, (43.6)	0.801
Ever smoker n, (%)	6, (18.2)	5, (12.8)	0.529
Disease duration (years, mean±std)	7.4±8.7	6.6±7.5	0.941
BMI (kg/m^2, mean±std)	22.0±4.2	22.4±3.4	0.696
Disease characteristics at baseline n, (%)			
A1 - Below 16 years	6, (18.2)	9, (23.1)	0.878
A2 - Between 17 and 40 years	19, (57.6)	21, (53.8)	
A3 - Above 40 years	8, (24.2)	9, (23.1)	
L1 - Ileal	24, (72.7)	22, (56.4)	0.356
L2 - Colonic	1, (3.0)	2, (5.1)	
L3 - Ileo-colonic	8, (24.2)	15, (38.5)	
L4 - Proximal disease	5, (15.2)	5, (12.8)	0.776
B1 - Non-stricturing, non-penetrating	16, (48.5)	15, (41.0)	0.797
B2 - Stricturing	8, (24.2)	10, (25.6)	
B3 - Penetrating	9, (27.3)	13, (33.3)	
Perianal disease	7, (21.2)	9, (23.1)	0.850
Past surgery	11, (33.3)	12, (30.8)	0.816
Extra-intestinal manifestations	6, (18.2)	5, (12.8)	0.529
Medical treatment n, (%)			
Naïve	19, (57.6)	20, (51.3)	0.167
Past therapy	0, (0)	4, (10.3)	
Current therapy	14, (42.4)	15, (38.5)	
Disease activity			
HBI (mean±std)	8.1±3.7	3.7±3.0	<0.001
CRP (mg/dl, mean±std)	2.2±1.6	1.7±2.4	0.032
Fcal (mg/kg, mean±std)	581±949	895±1257	0.220
Biomarker disease activity n,(%) [b]	13, (39.4)	23, (59.0)	0.098
SES-CD score (mean±std)	9.2±5.2	5.5±3.6	0.050
Rutgeerts score (mean±std)	2.0±2.0	2.1±1.4	0.921
Endoscopic disease activity n,(%) [c]	9, (27.3)	12, (30.8)	0.745
Indications and adaptations for the CDED n,(%)			
Alone	15, (45.5)	14, (35.9)	0.745
Adjunctive therapy	9, (27.3)	8, (20.5)	
Bridge therapy	7, (21.2)	11, (28.2)	
With steroids / antibiotic	2, (6.1)	6, (15.4)	
Partial enteral nutrition	14, (42.4)	11, (28.2)	0.207
Adherence to the CDED [d] n, (%)			
High adherence to the CDED	26, (78.8)	22, (56.4)	0.045

a Clinical response was determined among patients with active disease (by either clinical, biomarker or endoscopic criteria (n=72), as a drop of ≥3 HBI points
b Biomarker disease activity was defined as Fcal≥250 mg/kg d
c Endoscopic disease activity was defined as either SES-CD ≥ 7 or Rutgeerts score ≥ i2
d High adherence to the diet was defined as fairly/very adherence

Supplementary Table S3. Demographic and disease characteristics of patients with a clinically active disease allocated to the CDED alone compared to those treated with the CDED on top of other treatment options (n=48).

	**CDED alone (n=21)**	**CDED with other treatments [a] (n=27)**	**Pv**
Age (years, mean±std)	39.0±19.6	33.8±13.8	0.293
Gender – female n, (%)	12, (57.1)	16, (59.2)	0.760
Current smoker n, (%)	8, (38.0)	725.9)	0.963
Disease duration (years, mean±std)	5.4±9.5	8.6±8.1	0.215
BMI (kg/m^2, mean±std)	22.5±3.8	22.0±4.1	0.666
A1 - Below 16 years	1, (4.8)	7, (25.9)	0.149
A2 - Between 17 and 40 years	14, (66.7)	14, (51.9)	
A3 - Above 40 years	6, (28.6)	6, (22.2)	
L1 - Ileal	17, (81.0)	13, (48.1)	0.018
L2 - Colonic	1, (4.8)	0, (0.0)	
L3 - Ileo-colonic	3, (14.3)	14, (51.9)	
L4 - Proximal disease	4, (19.0)	4, (14.8)	0.696
B1 - Non-stricturing, non-penetrating	16, (76.2)	7, (25.9)	
B2 - Stricturing	4, (19.0)	8, (29.6)	0.001
B3 - Penetrating	1, (4.8)	12, (44.4)	
Perianal disease	1, (4.8)	12, (44.8)	0.002
Past surgery n, (%)	3, (14.3)	13, (48.1)	0.014
Extra-intestinal manifestations n, (%)	2, (9.5)	7, (25.9)	0.149
Partial enteral nutrition	6, (28.6)	11, (40.7)	0.382
Clinical response and remission rates			
Clinical remission rate[b] at week 6	17, (81.0)	13, (48.1)	0.020
Clinical remission rate[b] at week 12	17, (81.0)	13, (48.1)	0.020

[a] Patients treated with CDED and other treatments include those treated as an adjunctive therapy, bridge therapy, or with steroids/antibiotics.

[b] Clinical remission was defined as HBI<5 points

Supplementary Table S4. Demographic and disease characteristics of patients with a clinically active disease by follow-up duration with the CDED (n=48).

Follow-up duration	≤6 weeks (n=25)	12-24 weeks (n=21)	>24 weeks (n=2)	Pv
Age (years, mean±std)	40.1±16.4	33.2±17.3	30.0±11.3	0.349
Gender – female n, (%)	17, (68.0)	10, (47.6)	1, (50.0)	0.455
Current smoker n, (%)	6, (25.0)	3, (15.0)	0, (0.0)	0.837
Disease duration (years, mean±std)	8.5±8.6	5.4±9.2	9.7±13.0	0.490
BMI (kg/m^2, mean±std)	23.4±4.2	24.2±3.3	22.3±4.0	0.110
A1 - Below 16 years	6, (24.0)	1, (4.8)	1, (50.0)	
A2 - Between 17 and 40 years	11, (44.0)	16, (76.2)	1, (50.0)	0.123
A3 - Above 40 years	8, (32.0)	4, (19.0)	0, (0.0)	
L1 - Ileal	12, (48.0)	16, (76.2)	2, (100.0)	
L2 - Colonic	1, (4.0)	0, (0.0)	0, (0.0)	0.221
L3 - Ileo-colonic	12, (48.0)	5, (23.8)	0, (0.0)	
L4 - Proximal disease	4, (16.0)	4, (19.0)	0, (0.0)	0.781
B1 - Non-stricturing, non-penetrating	11, (44.0)	11, (52.4)	1, (50.0)	
B2 - Stricturing	7, (28.0)	4, (19.0)	1, (50.0)	0.805
B3 - Penetrating	7, (28.0)	6, (28.3)	0, (0.0)	
Perianal disease	8, (32.0)	5, (23.8)	0, (0.0)	0.559
Past surgery n, (%)	10, (41.7)	5, (25.0)	1, (50.0)	0.610
Extra-intestinal manifestations n, (%)	6, (25.0)	3, (15.0)	0, (0.0)	0.548
Clinical remission rate at week 6 [a]	14, (56.0)	14, (66.7)	2, (100.0)	0.047

[a] Clinical remission was defined as HBI<5 points
